# Fluorine-18 fluorodeoxyglucose positron emission tomography for cardiac sarcoidosis—is it time to consider a new radiotracer?

**DOI:** 10.1186/s13550-017-0322-z

**Published:** 2017-08-29

**Authors:** Georgios Christopoulos, Panithaya Chareonthaitawee

**Affiliations:** 1 Department of Internal Medicine, Mayo Clinic, 200 First Street SW, Rochester, 55905 MN USA; 2 Department of Cardiovascular Medicine, Mayo Clinic, 200 First Street SW, Rochester, MN 55905 USA

**Keywords:** Sarcoidosis, Positron emission tomography, Radiotracers, Fluorine-18 fluorodeoxyglucose, Fluorine-18 fluorothymidine

Cardiac fluorine-18 fluorodeoxyglucose (^18^F-FDG) positron emission tomography (PET) is increasingly used for detecting cardiac involvement and assessing the presence and severity of myocardial inflammation in sarcoidosis [1]. Numerous reports have highlighted the usefulness of ^18^F-FDG PET in improving the ability to identify and treat patients with this disease, and several societies now incorporate cardiac ^18^F-FDG PET findings as a diagnostic criterion for cardiac sarcoidosis [1]. Despite its increasing use, cardiac ^18^F-FDG PET has several limitations in identifying cardiac sarcoidosis. The main drawback relates to physiologic myocardial ^18^F-FDG uptake, which may occur under normal resting conditions in unaffected myocardium and poses challenges for the assessment of myocardial inflammation by ^18^F-FDG PET. Several approaches to suppress physiologic myocardial glucose uptake have been proposed. The majority advocate dietary preparations which include restricting carbohydrate intake while promoting high-fat consumption, prolonged fasting, heparin administration, or a combination of these approaches. However, a number of studies have demonstrated that, even with these approaches, physiologic uptake may not be completely suppressed. The reasons are multifold. First, low-carbohydrate, high-fat diets are very difficult to follow, and ensuring compliance is particularly problematic [2–4]. Second, it is virtually impossible to completely eliminate carbohydrate intake from the diet. Third, even in the absence of carbohydrate ingestion, glucose metabolism is active in tissues with high concentrations of glycogen, such as the myocardium. With respect to heparin administration in an effort to increase free fatty acid availability and suppress glucose metabolism [5], the majority of the studies demonstrate suppression of physiologic uptake with heparin use [5–9], although two studies suggest the converse [10, 11]. Heparin administration is also associated with increased bleeding risk and may not be appropriate in some patients. Another limiting factor to the use of ^18^F-FDG for assessment of cardiac sarcoidosis is that both inflammation and myocardial ischemia can increase glucose utilization and can further reduce the specificity of myocardial ^18^F-FDG uptake. Thus, an alternative tracer that does not require complicated dietary or fasting preparations and that has less nonspecific myocardial uptake would be highly useful for cardiac sarcoidosis. 3′-deoxy-3′-^18^F-fluorothymidine (^18^F-FLT), a promising PET tracer for evaluating tumor proliferative activity, has potential in this regard but has not yet been investigated in a systematic manner. In contrast to ^18^F-FDG, ^18^F-FLT uptake in normal myocardium is low even without prolonged fasting and/or a special diet prior to imaging.

In this issue of the journal, Norikane et al. [12] attempt to investigate the diagnostic accuracy of ^18^F-FLT in 20 patients with newly diagnosed cardiac or extracardiac sarcoidosis. The authors report that ^18^F-FLT was successful in detecting nearly all lesions (10 of 11 focal lesions) that were detected with ^18^F-FDG. This is the first study in the literature to directly compare the two tracers in patients with new untreated sarcoidosis.

The metabolism of ^18^F-FLT differs fundamentally from that of ^18^F-FDG since the former is part of DNA metabolism and therefore is a marker of tissue proliferation rather than glucose metabolism. As such, ^18^F-FLT PET has been mainly studied in the cancer population. However, ^18^F-FLT can also accumulate in granulomas, although to a lesser degree compared to tumor [13]. Thus far, the literature of ^18^F-FLT PET and its performance compared to ^18^F-FDG PET for sarcoidosis is limited to case reports [14, 15]. A recent study compared the standard uptake values (SUV) of ^18^F-FDG and ^18^F-FLT PET in 37 patients with biopsy-proven lymph node sarcoidosis and reported a mean SUV max of 12.7 with ^18^F-FDG and 6.0 with ^18^F-FLT. These findings are in line with the findings in the study by Norikane et al. [12].

Several strengths of the study by Norikane et al. [12] should be mentioned. The authors should be commended on adding to an area of the literature that is understudied. To the best of our knowledge, this is the first study to provide a head-to-head comparison of the two different tracers in patients with newly diagnosed untreated sarcoidosis. The number of patients is small but surpasses other studies, which have been limited to single-patient case reports. The authors used appropriate inclusion and exclusion criteria, and the diagnosis of sarcoidosis was made based on the updated 2016 guidelines. Quantitative data including standard uptake values were provided as part of the study. Finally, the authors performed a comprehensive review of the literature as part of their discussion.

Several limitations of the study by Norikane et al. [12] also warrant discussion. First, the authors did not use a high-fat, low-carbohydrate diet prior to their ^18^F-FDG PET studies. Based on the available literature, at least two high-fat, low-carbohydrate meals the day prior followed by fasting at least 4 to 12 h before the ^18^F-FDG PET study is preferred to prolonged fasting for suppressing physiologic myocardial ^18^F-FDG uptake and is the current recommended patient preparation by the joint expert consensus panel of the Society of Nuclear Medicine and Molecular Imaging (SNMMI) and the American Society of Nuclear Cardiology (ASNC) [1]. Thus, the ^18^F-FLT protocol is not being compared to an optimal gold standard. Similarly, the current PET protocol for cardiac sarcoidosis should include both resting myocardial perfusion and ^18^F-FDG images to differentiate the spectrum of cardiac sarcoidosis and improve the identification of cardiac involvement as recommended by the joint SNMMI-ASNC statement on the use of ^18^F-FDG PET/CT in cardiac sarcoidosis [1] and again the ^18^F-FLT protocol is not being compared to the optimal gold standard. A third limitation relates to the lack of data on whole-body imaging, which can impact diagnosis, prognosis, and guide biopsy, and management and is also recommended [1]. Furthermore, although this is the largest series of sarcoidosis patients to date, the sample size is small, and only 9 of the 20 patients had biopsy-proven extracardiac sarcoidosis; the rest of the patients were diagnosed clinically. None of the patients with cardiac sarcoidosis in the study had a positive endomyocardial biopsy, and only three patients actually underwent endomyocardial biopsy. While it is recognized that endomyocardial biopsy has low yield for cardiac sarcoidosis, histolologic correlation with ^18^F-FLT PET findings would be of interest and would provide great insight in this early stage of investigation of the tracer for this indication. All patients with cardiac sarcoidosis in this study were diagnosed on the basis of the revised Japanese Ministry of Health and Welfare Criteria, which requires a histologic or clinical diagnosis of extracardiac sarcoidosis, thereby limiting its utility in diagnosing isolated cardiac sarcoidosis [1]. Lastly, selection bias is a concern, as this appears to be a selected group of patients participating in a study.

Despite its limitations, the study by Norikane et al. [12] is a step in the right direction in finding alternative PET tracers for identifying cardiac sarcoidosis. Larger studies are needed to determine the accuracy and applicability of ^18^F-FLT PET as compared to ^18^F-FDG PET in cardiac and extracardiac sarcoidosis. Such studies should include the currently recommended protocol for ^18^F-FDG PET as mentioned above, to provide the optimal gold standard, and consider techniques to enhance the yield of endomyocardial biopsy to provide histologic correlation and better understanding of ^18^F-FLT uptake in this disease. Future studies should also examine the performance of ^18^F-FLT compared to ^18^F-FDG in monitoring the effect of treatment, particularly steroid therapy, which can alter the extracellular and intracellular glucose metabolism and affect ^18^F-FDG uptake. Those studies will offer the best comparative data between the two tracers and will help determine the best tracer for PET imaging in cardiac sarcoidosis.
